# Expanding Neurological Spectrum of Scrub Typhus: A Single-Center Case Series of Cerebral Venous Sinus Thrombosis, Opsoclonus-Myoclonus Syndrome, and Guillain-Barré Syndrome

**DOI:** 10.7759/cureus.111396

**Published:** 2026-06-23

**Authors:** Kumar Saurabh

**Affiliations:** 1 Department of Neurology, Orchid Medical Centre, Ranchi, IND

**Keywords:** cerebral venous sinus thrombosis, guillain–barré syndrome, neuroscrub typhus, opsoclonus–myoclonus syndrome, scrub typhus

## Abstract

Scrub typhus, caused by *Orientia tsutsugamushi*, is hyperendemic across the "tsutsugamushi triangle" and is increasingly recognized as a cause of diverse neurological complications. Although meningoencephalitis and seizures are the most frequently reported central nervous system manifestations, vascular, central autoimmune, and peripheral nervous system complications are increasingly being documented. Three mechanistically distinct neurological syndromes, cerebrovascular, central immune-mediated, and peripheral immune-mediated, can complicate scrub typhus. Each remains individually uncommon, and their co-occurrence at a single tertiary care center within a short temporal window is exceptional. Three patients with serologically confirmed scrub typhus, encountered within a short period at a single tertiary care center in eastern India, are described: (i) scrub typhus-associated cerebral venous sinus thrombosis (CVT) with hemorrhagic venous infarction in a young adult with concurrent severe vitamin B12 deficiency and hyperhomocysteinemia, representing a dual-hit prothrombotic state; (ii) scrub typhus-associated opsoclonus-myoclonus syndrome (OMS) presenting alongside meningoencephalitis and seizures, with opsoclonus and myoclonus becoming clinically prominent as sensorium improved; and (iii) scrub typhus-associated Guillain-Barré syndrome (GBS) presenting as an acute sensorimotor polyradiculoneuropathy with absent late responses and significant cerebrospinal fluid lymphocytic pleocytosis, suggesting overlapping inflammatory radiculitis. All three patients were diagnosed and managed successfully through early recognition, prompt initiation of doxycycline, and condition-specific therapy with anticoagulation, corticosteroids, and intravenous immunoglobulin, respectively, with favorable neurological outcomes. This series illustrates the broad pathophysiological repertoire of neuroscrub typhus and underscores the necessity of maintaining a high index of diagnostic suspicion in endemic regions.

## Introduction

Scrub typhus, caused by the obligate intracellular bacterium *Orientia tsutsugamushi*, is transmitted by the larval mite (chigger) of the trombiculid family and is hyperendemic across the "tsutsugamushi triangle" stretching from northern Japan to northern Australia and westward to the Indian subcontinent [1]. Although classically described as an acute febrile illness with eschar, lymphadenopathy, and multi-organ involvement, scrub typhus has emerged as a clinically important cause of neurological morbidity, with central nervous system involvement reported in approximately 12%-26% of hospitalized adults [2,3].

The pathophysiology of neurological injury in scrub typhus is multifactorial. Direct endothelial invasion triggers small-vessel vasculitis, while immune-mediated mechanisms, including cytokine release, autoantibody formation, and molecular mimicry, amplify the resultant tissue injury [1,2]. The cumulative effect of endothelial dysfunction, microcirculatory disturbance, and immune dysregulation accounts for the wide neurological spectrum observed. These manifestations are best considered by the level of the neuraxis involved: central nervous system involvement, comprising meningitis, encephalitis, seizures, cerebellitis, myelitis, movement disorders, and cerebrovascular events such as ischemic stroke and cerebral venous sinus thrombosis (CVT); peripheral nervous system involvement, comprising cranial neuropathies, Guillain-Barré syndrome (GBS), and other peripheral neuropathies; and muscle involvement, in the form of myositis and, occasionally, rhabdomyolysis with creatine kinase elevation [2,3].

Three particular syndromes lie at the rarer end of this spectrum. Scrub typhus-associated CVT reflects the prothrombotic consequences of rickettsial endothelial injury; only a small number of cases have been documented globally, and coexistence with additional thrombophilic states such as hyperhomocysteinemia is exceptional [4-6]. Opsoclonus-myoclonus syndrome (OMS), an immune-mediated disorder of cerebellar and brainstem ocular motor circuitry, has been described in only a small number of scrub typhus patients worldwide [7,8]; in the largest reported series, a recent Indian retrospective cohort identified opsoclonus in just 18 of 1,650 scrub typhus patients, with even fewer fulfilling criteria for full OMS [9]. GBS is similarly uncommon in this context, with only a limited number of cases reported globally [10-13].

Three serologically confirmed cases of scrub typhus presenting with these three distinct rare neurological syndromes are described, all encountered within a short period at a single tertiary care center in eastern India. The simultaneous occurrence of cerebrovascular, central immune-mediated, and peripheral immune-mediated manifestations within one institutional cohort is, to my knowledge, previously unreported and offers a unique opportunity to examine the mechanistic breadth of neuroscrub typhus.

## Case presentation

Case 1: Scrub typhus-associated CVT with hemorrhagic venous infarction in a setting of severe hyperhomocysteinemia

A 28-year-old previously healthy male presented with one week of high-grade intermittent fever followed by acute-onset left-sided weakness and a single generalized tonic-clonic seizure. On admission, he was conscious, oriented, and hemodynamically stable, with dense left hemiplegia (power 0/5) and no meningismus.

**Figure 1:**
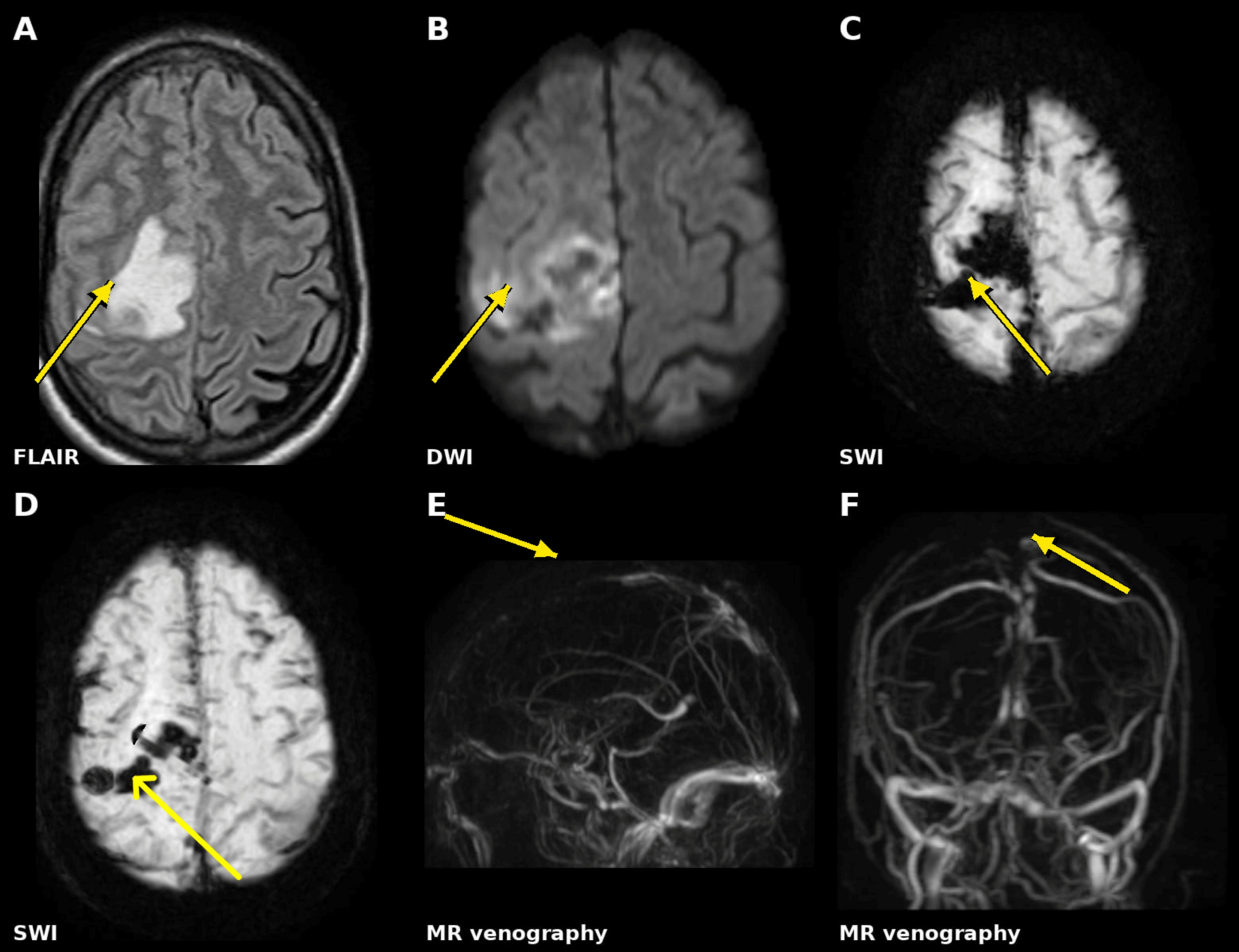
Multimodal magnetic resonance imaging demonstrating scrub typhus-associated superior sagittal sinus thrombosis with hemorrhagic venous infarction. Axial FLAIR (A, yellow arrow) shows a hyperintense lesion in the right parasagittal fronto-parietal region consistent with a venous infarct. Diffusion-weighted imaging (B, arrow) demonstrates corresponding hyperintensity, confirming acute injury. Susceptibility-weighted imaging at two contiguous axial levels (C and D, arrows) shows extensive blooming with discrete, rounded hypointense foci within the venous infarct, representing intralesional hemorrhage. Lateral (E, arrow) and coronal (F, arrow) MR venography demonstrate non-visualization of the superior sagittal sinus with prominent collateral cortical veins, confirming sinus thrombosis. FLAIR, fluid-attenuated inversion recovery; DWI, diffusion-weighted imaging; SWI, susceptibility-weighted imaging; MR, magnetic resonance; SSS, superior sagittal sinus.

Magnetic resonance imaging demonstrated diffusion restriction with susceptibility-weighted blooming in the right parasagittal frontoparietal region, consistent with hemorrhagic venous infarction. MR venography revealed nonvisualization of the superior sagittal sinus, confirming thrombosis (Figure 1). 

Laboratory evaluation revealed an elevated C-reactive protein level, neutrophilic leukocytosis, an elevated erythrocyte sedimentation rate, scrub typhus IgM positivity by enzyme-linked immunosorbent assay, macrocytic anemia, severe vitamin B12 deficiency, and markedly elevated serum homocysteine (Table 1). A formal inherited thrombophilia panel (protein C, protein S, antithrombin) was not pursued, as an acquired prothrombotic state had already been established, namely severe vitamin B12 deficiency with marked hyperhomocysteinemia, and such assays are unreliable when sampled during acute thrombosis and while on anticoagulation. The patient was therefore diagnosed with scrub typhus-associated CVT with hemorrhagic venous infarction in the setting of severe hyperhomocysteinemia, reflecting a synergistic dual-hit prothrombotic state of rickettsial endothelial injury and metabolic thrombophilia. 

**Table 1:** Summary of investigations: Case 1 (scrub typhus-associated cerebral venous sinus thrombosis) CVT, cerebral venous sinus thrombosis; MRI, magnetic resonance imaging; FLAIR, fluid-attenuated inversion recovery; DWI, diffusion-weighted imaging; SWI, susceptibility-weighted imaging; MR, magnetic resonance; IgM, immunoglobulin M; ELISA, enzyme-linked immunosorbent assay; MCV, mean corpuscular volume; OD, optical density.

Investigation	Result	Reference range
MRI brain	Hemorrhagic venous infarction (right parasagittal fronto-parietal)	Not applicable
MR venography	Non-visualization of the superior sagittal sinus	Not applicable
Scrub typhus IgM (ELISA)	Reactive (OD 1.12)	Non-reactive (OD ≤0.46)
C-reactive protein	62.1 mg/L	<5.0 mg/L
Total leukocyte count	12.11 × 10⁹/L	4.0–11.0 × 10⁹/L
Erythrocyte sedimentation rate	85 mm/h	<15 mm/h
Hemoglobin	12.57 g/dL	13.5–17.5 g/dL
Mean corpuscular volume	108.2 fL	80–96 fL
Platelet count	347 × 10⁹/L	150–410 × 10⁹/L
Serum vitamin B12	<50 pg/mL	120–914 pg/mL
Serum homocysteine	>50 µmol/L	5.46–16.20 µmol/L
Serum creatinine	0.77 mg/dL	0.72–1.18 mg/dL
Final diagnosis	Scrub typhus–associated CVT with hemorrhagic venous infarction and severe hyperhomocysteinemia	

He received doxycycline 100 mg twice daily, therapeutic low-molecular-weight heparin transitioned to warfarin, parenteral vitamin B12, and antiepileptic therapy. Fever resolved within 72 hours of doxycycline initiation. Anticoagulation was continued in keeping with international CVT guidelines despite the intracranial hemorrhage [5]. Serial imaging showed no progression of hemorrhage, and the patient was discharged neurologically stable with residual left-sided weakness and a structured rehabilitation plan.

On structured neurological follow-up at three months, the patient had improved progressively and was independently mobile without any significant limp. Left upper limb power had returned to 5/5 across all major muscle groups. In the left lower limb, dorsiflexion at the ankle remained mildly weak (4/5), while all other muscle groups had recovered to 5/5. Anticoagulation, vitamin B12 supplementation, and antiepileptic therapy were continued, and the patient resumed activities of daily living without restriction.

Case 2: Scrub typhus-associated OMS with meningoencephalitis and seizures

A 33-year-old woman presented with 10 days of fever and headache followed by multiple generalized tonic-clonic seizures and impaired sensorium on the day of admission. On examination, she was drowsy (Glasgow Coma Scale E3V3M5) without focal neurological deficit [14]. She required intensive care admission with antiepileptic therapy and empirical antimicrobial coverage for meningoencephalitis.

Initial systemic evaluation revealed a markedly elevated C-reactive protein, transaminitis, acute kidney injury, and microcytic anemia, all of which were managed with appropriate supportive care. Cerebrospinal fluid analysis demonstrated lymphocytic pleocytosis (18 cells/µL, 90% lymphocytes) with protein at the upper limit of normal and no hypoglycorrhachia; cerebrospinal fluid, blood, and urine cultures were sterile; cerebrospinal fluid Gram stain, acid-fast stain, and India ink preparation showed no organisms; and cryptococcal antigen, GeneXpert *Mycobacterium tuberculosis*, dengue, malaria, and leptospira screens were all negative. Scrub typhus IgM was reactive on a rapid immunochromatographic card. Contrast magnetic resonance imaging of the brain was unremarkable, with no parenchymal lesion, diffusion restriction, or meningeal enhancement (Tables 2, 3). 

**Table 2:** Summary of serum and blood investigations: Case 2 (scrub typhus-associated opsoclonus-myoclonus syndrome with meningoencephalitis) Values are at presentation; the C-reactive protein, transaminases, and renal indices normalized with treatment (peak creatinine 3.52 mg/dL). MCV, mean corpuscular volume; TLC, total leukocyte count; ESR, erythrocyte sedimentation rate; AST, aspartate aminotransferase; ALT, alanine aminotransferase; LDH, lactate dehydrogenase; NS1, nonstructural protein 1; IgM, immunoglobulin M; IgG, immunoglobulin G; HIV, human immunodeficiency virus; HBsAg, hepatitis B surface antigen; MRI, magnetic resonance imaging.

Investigation	Result	Reference range
C-reactive protein	99.7 mg/L	<5.0 mg/L
Hemoglobin	9.94 g/dL	12–16 g/dL
Mean corpuscular volume	77.6 fL	80–96 fL
Total leukocyte count	10.81 × 10⁹/L	4.0–11.0 × 10⁹/L
Platelet count	155 × 10⁹/L	150–410 × 10⁹/L
Erythrocyte sedimentation rate	80 mm/h	<20 mm/h
Aspartate aminotransferase (AST)	137.5 U/L	0–35 U/L
Alanine aminotransferase (ALT)	87.1 U/L	0–35 U/L
Lactate dehydrogenase (LDH)	708.6 U/L	0–247 U/L
Serum urea	57.5 mg/dL	17–43 mg/dL
Serum creatinine	2.17 mg/dL	0.55–1.02 mg/dL
Dengue NS1 antigen/IgM/IgG	Negative	Negative
Malaria rapid antigen	Negative	Negative
Leptospira IgM/IgG (rapid card)	Non-reactive	Non-reactive
Serum HIV/hepatitis C/HBsAg	Non-reactive	Non-reactive
Scrub typhus IgM (rapid card)	Reactive for IgM	Non-reactive
MRI brain (contrast)	Unremarkable	Not applicable
Final diagnosis	Scrub typhus-associated opsoclonus-myoclonus syndrome with meningoencephalitis and seizures	

**Table 3:** Cerebrospinal fluid analysis: Case 2 CSF, cerebrospinal fluid; ADA, adenosine deaminase; MTB/RIF, Mycobacterium tuberculosis/rifampicin-resistance assay.

Investigation	Result	Reference range
CSF total cell count	18 /µL	0–5/µL
CSF lymphocyte proportion	90%	—
CSF protein	38.5 mg/dL	15–45 mg/dL
CSF glucose	132.9 mg/dL	40–70 mg/dL
CSF adenosine deaminase (ADA)	1.4 U/L	<10 U/L
CSF Gram/acid-fast/India-ink stain	No organisms seen	No organisms
CSF, blood, and urine cultures	Sterile	No growth
CSF cryptococcal antigen	Not detected	Not detected
CSF GeneXpert MTB/RIF	Not detected	Not detected
Interpretation	Lymphocytic pleocytosis with protein at the upper normal limit and no hypoglycorrhachia; sterile cultures with negative stains and antigen testing exclude pyogenic, tuberculous, and cryptococcal meningitis	—

As the patient's sensorium began to improve, conjugate involuntary saccadic eye movements, together with truncal, postural, and appendicular myoclonus on attempting to sit up or raise the arms, became evident (Video 1). A clinical diagnosis of scrub typhus-associated OMS with meningoencephalitis was made [7,8].

**Video 1:** Opsoclonus-myoclonus in the patient with scrub typhus-associated opsoclonus-myoclonus syndrome (Case 2), recorded as sensorium improved. The recording demonstrates conjugate involuntary saccadic eye movements (opsoclonus), together with irregular, arrhythmic myoclonic jerks of the trunk and limbs accentuated on maintaining an upright posture and on extending the arms. To aid visualization of the eye movements, the ocular region in the relevant segment has been digitally magnified and is shown at reduced (half) playback speed; the earlier portion of the clip is shown at original scale and speed.

The patient was started on doxycycline, intravenous corticosteroids, and continued antiepileptic therapy. Intravenous immunoglobulin was advised but could not be administered owing to financial constraints. Over the subsequent two weeks, sensorium normalized, opsoclonus and myoclonus reduced substantially, and the patient progressed from a bed-bound state to sitting unsupported (Video 2) and accepting oral feeds independently before discharge.

**Video 2:** Clinical improvement in the same patient (Case 2) during the hospital course. The previously prominent myoclonus has markedly reduced, and the patient is able to sit independently.

Case 3: Scrub typhus-associated GBS with lymphocytic pleocytosis

A 61-year-old man was admitted to internal medicine with approximately 20 days of high-grade fever and severe myalgia. Scrub typhus IgM serology returned positive, and doxycycline was initiated. After about one week of hospitalization, he developed rapidly progressive ascending weakness, beginning in one lower limb and extending sequentially to involve the contralateral lower limb and both upper limbs over three to four days. There was no bulbar involvement, sensory level, autonomic dysfunction, or radicular pain. He was transferred to the neurology unit.

Examination revealed flaccid quadriparesis with absent deep tendon reflexes in the lower limbs and sluggish reflexes in the upper limbs, bilateral flexor plantar responses, and mild distal sensory impairment. Sensorium remained intact. There was no bulbar weakness or respiratory compromise at presentation (Table 4). 

**Table 4:** Summary of investigations: Case 3 (scrub typhus-associated GBS) Acute kidney injury was present at admission (peak creatinine 2.76 mg/dL, peak urea 95.9 mg/dL) and recovered fully with supportive care. GBS, Guillain-Barré syndrome; Hb, hemoglobin; TLC, total leukocyte count; ESR, erythrocyte sedimentation rate; AST, aspartate aminotransferase; MRI, magnetic resonance imaging; IVIG, intravenous immunoglobulin; IgM, immunoglobulin M; ELISA, enzyme-linked immunosorbent assay; OD, optical density.

Investigation	Result	Reference range
Hemoglobin	10.82 g/dL	13.5–17.5 g/dL
Total leukocyte count	4.01 × 10⁹/L	4.0–11.0 × 10⁹/L
Platelet count	192 × 10⁹/L	150–410 × 10⁹/L
Erythrocyte sedimentation rate	70 mm/h	<15 mm/h
Aspartate aminotransferase (AST)	69 U/L (peak, improving)	0–35 U/L
Serum urea (peak)	95.9 mg/dL	17.0–43.0 mg/dL
Serum creatinine (peak)	2.76 mg/dL	0.72–1.18 mg/dL
Scrub typhus IgM (ELISA)	Reactive (OD 7.31)	Non-reactive (OD ≤0.46)
MRI whole spine (contrast)	No nerve-root or leptomeningeal enhancement	Not applicable
Cerebrospinal fluid analysis	See Table 5	See Table 5
Nerve conduction study	See Table 6	See Table 6
Final diagnosis	Scrub typhus–associated Guillain–Barré syndrome (acute sensorimotor polyradiculoneuropathy with absent late responses)	

Cerebrospinal fluid analysis demonstrated markedly elevated protein with significant lymphocytic pleocytosis, a pattern atypical for classical GBS and suggestive of overlapping inflammatory radiculitis (Table 5). Given the degree of pleocytosis, alternative infectious and neoplastic causes of polyradiculitis were actively excluded: serum viral markers (HIV, hepatitis B, and hepatitis C) were nonreactive, CSF herpes simplex virus 1/2 PCR was negative, and CSF cytology showed no malignant cells. Magnetic resonance imaging of the whole spine with contrast revealed no nerve root enhancement, leptomeningeal enhancement, or compressive pathology. 

**Table 5:** Cerebrospinal fluid analysis: Case 3 CSF, cerebrospinal fluid; ADA, adenosine deaminase; HSV, herpes simplex virus; PCR, polymerase chain reaction; HIV, human immunodeficiency virus; GBS, Guillain–Barré syndrome.

Parameter	Result	Reference range
Protein	160 mg/dL	15–45 mg/dL
Total cell count	120 /µL	0–5 /µL
Lymphocyte proportion	85%	—
Glucose	58 mg/dL	40–70 mg/dL
Adenosine deaminase (ADA)	4.4 U/L	<10 U/L
Herpes simplex virus 1/2 PCR	Negative	Negative
Cytology	No malignant cells	No malignant cells
Serum HIV / hepatitis B / hepatitis C	Non-reactive	Non-reactive
Interpretation	Elevated protein with lymphocytic pleocytosis, atypical for classical GBS; mimics excluded — consistent with overlapping inflammatory radiculitis	

Nerve conduction studies were performed serially. An initial lower-limb study showed bilateral S1 radiculopathy with preserved F-wave latencies. A subsequent study of all four limbs (Table 6) showed preserved distal motor latencies and conduction velocities, with largely preserved compound muscle action potential (CMAP) amplitudes apart from a reduced right ulnar CMAP. Ulnar sensory nerve action potential (SNAP) amplitudes were reduced bilaterally, whereas median and sural SNAP were preserved. F-waves were absent in the median, ulnar, peroneal, and tibial nerves, with bilaterally absent soleus H-reflexes. This predominant loss of late responses, accompanied by reduced ulnar motor and sensory amplitudes with otherwise preserved distal motor conduction, was consistent with early evolving GBS [15,16]. 

**Table 6:** Nerve conduction study: Case 3 (study of all four limbs) NCS, nerve conduction study; CMAP, compound muscle action potential; SNAP, sensory nerve action potential.

Study/parameter	Right	Left	Reference range
Motor nerve studies			
Median, distal motor latency (ms)	3.02	4.69	≤4.4
Median, CMAP amplitude (mV)	7.2	5.4	≥4.0
Median, conduction velocity (m/s)	53.3	51.1	≥49
Ulnar, distal motor latency (ms)	3.23	3.02	≤3.3
Ulnar, CMAP amplitude (mV)	4.4 (reduced)	6.2	≥6.0
Ulnar, conduction velocity (m/s)	55.2	49.0	≥49
Common peroneal, distal motor latency (ms)	5.10	5.94	≤6.5
Common peroneal, CMAP amplitude (mV)	2.7	2.7	≥2.0
Common peroneal, conduction velocity (m/s)	44.1	45.3	≥41
Posterior tibial, distal motor latency (ms)	4.48	5.21	≤6.0
Posterior tibial, CMAP amplitude (mV)	7.1	4.9	≥4.0
Posterior tibial, conduction velocity (m/s)	49.6	46.0	≥41
Sensory nerve studies			
Median, SNAP amplitude (µV)	53.6	40.6	≥20
Median, conduction velocity (m/s)	48.6	52.6	≥50
Ulnar, SNAP amplitude (µV)	6.8 (reduced)	4.0 (reduced)	≥17
Ulnar, conduction velocity (m/s)	38.5 (reduced)	44.5	≥50
Sural, SNAP amplitude (µV)	7.5	8.7	≥6
Sural, conduction velocity (m/s)	50.7	50.0	≥40
Late responses			
F-wave (median, ulnar, peroneal, tibial)	Absent	Absent	Present
H-reflex (soleus)	Absent	Absent	Present
Impression: generalized symmetric sensorimotor polyradiculoneuropathy with absent late responses, consistent with Guillain–Barré syndrome.			

The patient was treated with intravenous immunoglobulin at 0.4 g/kg/day for five days, alongside continued doxycycline and supportive care. Over the following week, motor strength improved substantially. No autonomic instability, cranial nerve involvement, or requirement for mechanical ventilation developed. He was discharged in a stable condition with continued rehabilitation.

## Discussion

The three cases described here illustrate the breadth of neurological involvement that may complicate scrub typhus and, more importantly, the mechanistic diversity underlying this spectrum. While meningoencephalitis and seizures remain the most frequently encountered manifestations [2,3], the present series captures three rarer phenotypes: a thrombotic cerebrovascular event, a central immune-mediated movement disorder, and a peripheral immune-mediated polyradiculoneuropathy, each representing a distinct pathway by which scrub typhus can disturb the nervous system.

The shared upstream driver across these phenotypes appears to be the host immune-inflammatory response to Orientia tsutsugamushi. The organism exhibits tropism for vascular endothelium and produces a generalized small-vessel vasculitis, while parallel immune dysregulation, including cytokine elaboration, complement activation, autoantibody formation, and molecular mimicry, amplifies tissue injury [1,2]. Downstream, the dominant pathway then determines the phenotype: endothelial injury with a prothrombotic milieu in CVT, and postinfectious autoimmunity in the central (OMS) and peripheral (GBS) immune-mediated syndromes.

Cerebral venous sinus thrombosis

CVT is an uncommon cause of stroke in young adults, accounting for approximately 0.5%-1% of all cerebrovascular events [5]. Scrub typhus-associated CVT remains rare, with only isolated cases reported worldwide [6]. The proposed mechanism involves rickettsia-induced endothelial injury, capillary leak, perivasculitis, and a hypercoagulable milieu driven by elevated inflammatory cytokines [1,2]. The added thrombophilic burden of hyperhomocysteinemia, an established independent risk factor for venous thrombosis owing to its adverse effects on endothelial nitric oxide bioavailability, oxidative stress, and platelet aggregation [17], is biologically synergistic, and the concordant macrocytic anemia in this patient supports the underlying vitamin B12 deficiency. To my knowledge, the coexistence of scrub typhus, severe vitamin B12 deficiency, and hyperhomocysteinemia in a single CVT case has not previously been emphasized. Therapeutic anticoagulation in the presence of intracranial hemorrhage, while initially counterintuitive, is supported by international consensus guidelines [5,18] and was followed by clinical and radiological stability in this patient.

Opsoclonus-myoclonus syndrome

OMS is characterized by chaotic, conjugate, multidirectional saccadic eye movements (opsoclonus), generalized myoclonus, and ataxia. The pathophysiology is presumed to involve immune-mediated dysfunction of cerebellar-brainstem ocular motor circuitry, with disinhibition of the brainstem saccadic burst generators that govern conjugate eye movements. Although classically described as a paraneoplastic syndrome (neuroblastoma in children, small-cell lung carcinoma in adults), parainfectious OMS is now increasingly recognized [7,8]. In scrub typhus, OMS remains exceedingly rare, with only a small number of individual case reports published [7,8]. The largest available cohort identified opsoclonus in only 18 of 1,650 scrub typhus patients, with even fewer fulfilling full OMS criteria [9]. Importantly, neuroimaging is typically unremarkable, as in this patient, favoring a functional rather than structural pathology. The coexistence of meningoencephalitis, seizures, multi-organ dysfunction, and OMS in the same patient is, to my knowledge, particularly uncommon. Optimal management combines antimicrobial therapy with immunomodulation, principally corticosteroids and intravenous immunoglobulin [8]. This patient demonstrated meaningful recovery on corticosteroids alone, illustrating that the limitations of resource-constrained settings need not preclude favorable outcomes when therapy is initiated promptly.

Guillain-Barré syndrome

GBS is a heterogeneous immune-mediated polyradiculoneuropathy classically triggered by antecedent infection. While *Campylobacter jejuni* and cytomegalovirus remain the best-characterized antecedents, scrub typhus has been increasingly implicated, with only a limited number of cases reported globally, including pediatric presentations [10-13,19,20]. In this patient, the electrophysiological picture evolved from an early bilateral S1 radiculopathy to a generalized sensorimotor polyradiculoneuropathy with absent late responses; with largely preserved distal motor amplitudes and conduction velocities, this pattern was consistent with an early, evolving GBS. The marked CSF lymphocytic pleocytosis, atypical for classical GBS, raises an important diagnostic consideration: dual pathology comprising both direct infection-related radiculomeningeal inflammation and a postinfectious immune-mediated neuropathy. This concept of overlapping inflammatory radiculitis with electrophysiologically supported GBS in scrub typhus has been described in only a small number of reports [19,21]. The absence of contrast enhancement on MRI of the spine does not exclude GBS, as nerve root enhancement is inconsistent and may be absent even in confirmed cases. This patient's brisk response to intravenous immunoglobulin reinforces the utility of standard GBS therapy even when CSF findings are atypical.

Synthesis

Taken together, these three cases reinforce several key clinical principles. First, scrub typhus must be considered in any unexplained febrile neurological illness in endemic regions, regardless of the specific neurological phenotype. Second, the mechanistic spectrum of neurological involvement is broad and extends well beyond the more familiar manifestations of meningoencephalitis and seizures. Third, despite the diagnostic complexity of these rare syndromes, outcomes are favorable when scrub typhus is recognized early and condition-specific therapy, namely anticoagulation, immunomodulation, or intravenous immunoglobulin, is added to timely antimicrobial coverage. A multidisciplinary approach involving neurology, internal medicine, critical care, and rehabilitation services is central to optimal outcomes.

The simultaneous occurrence of these three rare syndromes within a short period at a single center is striking and may reflect either a true seasonal epidemiological cluster or, more probably, increased recognition and serological testing in a hyperendemic region. Either explanation argues for greater clinical awareness and routine inclusion of scrub typhus serology in the diagnostic workup of acute and subacute neurological illness in endemic settings.

## Conclusions

Scrub typhus is associated with a wider and more heterogeneous neurological spectrum than is often appreciated. The present series, describing CVT, OMS, and GBS in three patients managed at a single tertiary care center within a short period, illustrates the cerebrovascular, central immune-mediated, and peripheral immune-mediated phenotypes through which scrub typhus may manifest. Early recognition, prompt initiation of doxycycline, and timely condition-specific therapy resulted in favorable outcomes in all three patients. For neurologists and physicians practicing in endemic regions, scrub typhus warrants active consideration in the differential diagnosis of unexplained neurological illness, and routine serological evaluation may help identify cases that would otherwise be misclassified.
